# Quantum Mechanical Behavior of Hydrogen Bonds Enables Supramolecular Structure in a Weak Acid–Base Monoprotic Complex

**DOI:** 10.1021/jacs.4c17870

**Published:** 2025-04-09

**Authors:** Anit Gurung, Rui Zhang, Lu Wang, Daniel G. Kuroda

**Affiliations:** † Department of Chemistry, Louisiana State University, Baton Rouge, Louisiana 70803, United States; ‡ Department of Chemistry and Chemical Biology, Institute for Quantitative Biomedicine, Rutgers University, Piscataway, New Jersey 08854, United States

## Abstract

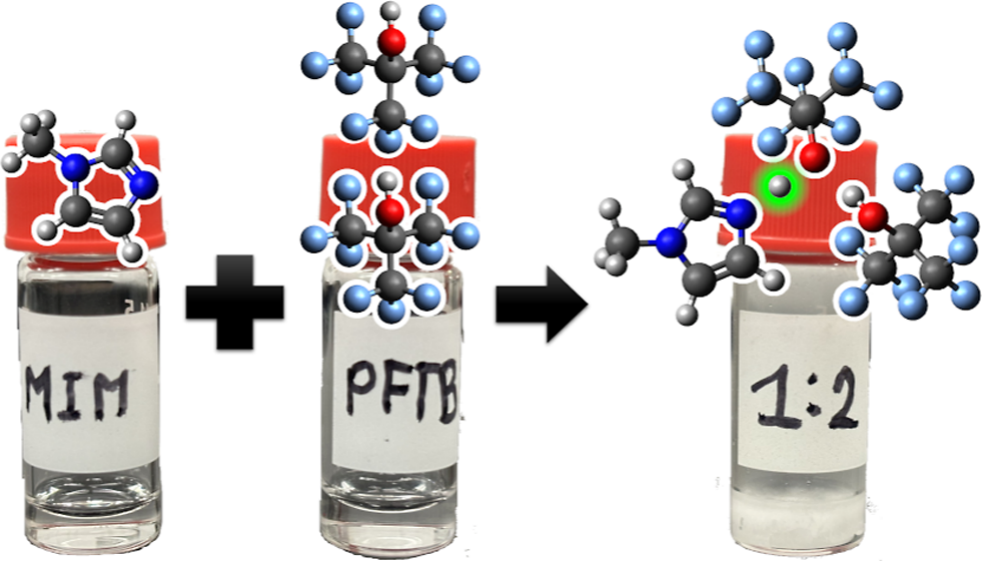

The unconventional supramolecular chemistry between perfluoro-*tert*-butanol (PFTB), as an acid, and 1-methylimidazole (MIM), as a base, is presented. Supramolecular chemistry occurs in MIM-PFTB mixtures with a base-to-acid molar ratio of 1:2, or higher, and coincides with the formation of strong hydrogen bonds (SHBs) in which the acidic hydrogen atoms are quantum mechanically delocalized. Evidence for the SHB and the hydrogen atom sharing is obtained from IR and ^1^H NMR spectroscopies and X-ray crystallography. First-principles simulations incorporating both electronic and nuclear quantum effects verify the presence of the SHBs and demonstrate that the broad IR absorption band centered at 2400 cm^–1^ and the large downfield ^1^H NMR of the complexes are a consequence of the hydrogen atom sharing between the acid and the base. The supramolecular behavior reported for PFTB-MIM has not been previously observed in other monoprotic acid–base mixtures forming either conventional or SHBs. Hence, MIM-PFTB mixtures depart from the behavior typically exhibited by other liquid mixtures, demonstrating that electronic and nuclear quantum effects play an important role in driving the unconventional supramolecular chemistry observed in MIM-PFTB samples.

## Introduction

**Figure 1:**
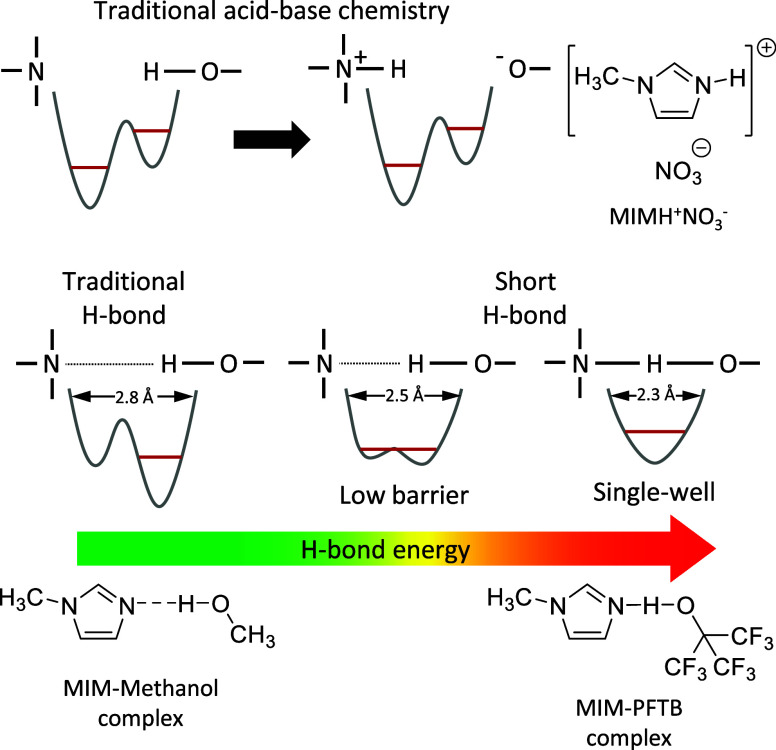
Schematic representation of the possible acid–base interactions and their representative molecular systems, including the traditional proton transfer reaction (upper panel) and the formation of a hydrogen bond (bottom panel), where N and O represent the heteroatoms of the base and the acid, respectively. The bottom panel also showcases the quantum mechanical states created as a function of the strength of the hydrogen bond. Horizontal red lines represent the zero-point energy of the O–H stretching mode.

The importance of quantum mechanical effects in acid–base reactions was first recognized by Bell a century ago.^1^ Because of their light weight, protons have a non-negligible probability of passing through large energy barriers, such as those involved in the proton transfer of acid–base reactions. This effect is usually referred to as the quantum tunneling effect.^1−5^ Another quantum mechanical property of the hydrogen atom is its delocalization between an acid and a base, forming a hydrogen bond. Hydrogen atom delocalization has been shown to play a significant role in short hydrogen bonds, as the zero-point energy of the hydrogen shuttling becomes comparable to or greater than the barrier to proton transfer.^6−12^ Traditional acid–base chemistry, first introduced by Bronsted–Lowry (BL),^13,14^ proposes that the proton of an acid (HA) is transferred to a base (B) to form their conjugate ion pairs (i.e., A^–^ and HB^+^) when the difference in acid dissociation constant (Δp*K*_a_) between the acid and the conjugated acid of the base is sufficiently large (Figure 1). BL theory has provided an excellent description of the acid–base chemistry in aqueous solutions, where the proton transfer barrier is decreased as water molecules stabilize the ionized states.^15^ However, this theory does not properly describe systems where the zero-point energy is comparable to or larger than the barrier (Figure 1). The latter category includes binary mixtures of a weak acid and a weak base in nonaqueous environments, which do not always result in a proton transfer but often in the formation of a hydrogen-bonded complex in which the hydrogen atom can be quantum mechanically delocalized between the two heteroatoms (Figure 1).^16,17^

The hydrogen-bonded complexes between an acid and a base, in which the hydrogen atom can be bonded to both the acid and the base due to the quantum mechanical position uncertainty, are highly unconventional and have distinct IR and NMR signatures.^7−9,17−22^ In particular, complexes with strong hydrogen bonds (SHBs)^23^ often exhibit a large downfield change in the chemical shift of the acidic hydrogen atom due to a substantial deshielding effect.^7,9,19−21^ In addition, the O–H or N–H stretch frequency is significantly broadened and red-shifted by more than 500 cm^–1^.^7,17,21,24^ Classical chemistry theories^13,14,25,26^ cannot fully explain these features because they do not include the quantum nature of the hydrogen atom.^5,11,12,17,27−30^ The small mass of the hydrogen atom emphasizes the importance of including nuclear quantum effects, such as zero-point energy and tunneling, to describe the behavior of the hydrogen atom in these acid–base complexes.^11,31^

First-principles simulations have revealed that both nuclear and electronic quantum effects govern the structure and energetics of short hydrogen bonds in the condensed phase.^10,11,17,30,32−34^ Moreover, only when the quantum nature of the acidic hydrogen atom, in both the electronic and nuclear degrees of freedom, is taken into consideration, the delocalization of the hydrogen atom is observed in the simulations.^17^ An example of such behavior has been observed in the complexes formed by acetic acid (HAc) and 1-methylimidazole (MIM), with relatively low Δp*K*_a_, at various molar ratios.^17^ As the nonaqueous Δp*K*_a_ approaches zero, these mixtures defy the traditional acid–base chemistry theories because the resulting systems do not result in ionic compounds but in complexes SHBs, in which the acidic hydrogen atoms are delocalized.^17^ As a consequence, these complexes present a broad IR absorption band in the 2250–3000 cm^–1^ region and a large downfield of the ^1^H NMR chemical shift between 12 and 16 ppm, in agreement with their unconventional acid–base interaction.

**Figure 2:**
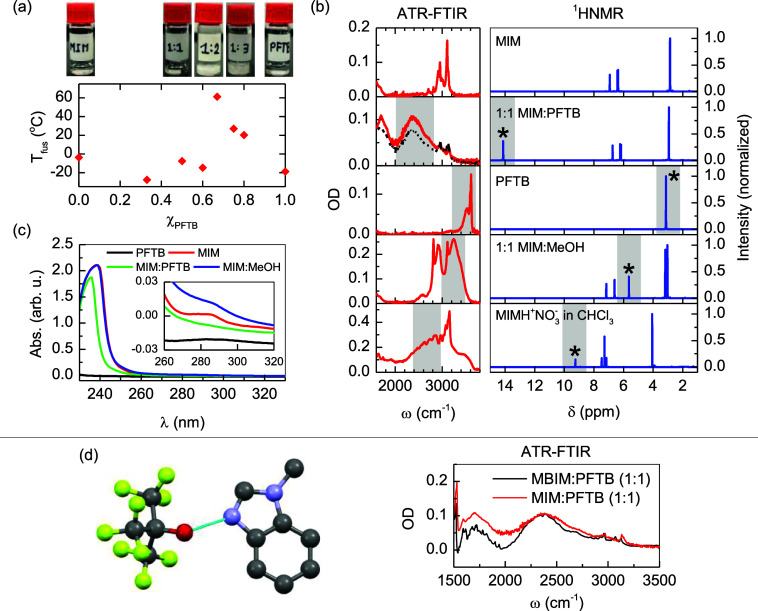
(a) Physical state and fusion temperature as a function of the PFTB concentration in different MIM-PFTB mixtures. (b) IR and ^1^H NMR spectroscopic signatures for MIM, PFTB, and various MIM-containing mixtures. In the IR spectra, the binary mixtures of MIM and PFTB are presented with the 1:1 *r*_M_ in red and the 1:2 *r*_M_ dashed black. In the ^1^H NMR spectra, stars denote the signals of the acidic proton. Gray areas denote the spectroscopic signatures of the O–H group (c) UV–vis spectra for MIM, PFTB, their 1:1 mixture, and the MIM-methanol mixture. (d) X-ray crystal structure for the MBIM-PFTB supramolecular complex and its corresponding IR spectra.

Here, another unconventional acid–base pair is presented. The pair consists of MIM, a weak base, and perfluorinated tert-butanol (PFTB), a weak acidic alcohol with an aqueous p*K*_a_ of 5.4 (Figure 1).^35^ Since PFTB is a weaker acid than HAc (p*K*_a_ of 4.8), the Δp*K*_a_ of the MIM-PFTB pair is closer to zero^36^ and explains the formation of SHBs in these mixtures as well. However, the MIM-HAc mixtures are liquids at all molar ratios, contrasting with the MIM-PFTB mixtures at molar ratios (*r*_M_) of 1:2 and higher, which form stable solids at room temperature (Figure 2a). The formation of these MIM-PFTB solid complexes deviates from the solid salt formation expected to occur at a 1:1 molar ratio in traditional acid–base chemistry for the neutralization reaction between a monoprotic acid and a monoprotic base. Overall, the most notable feature of the monoprotic MIM-PFTB system is the formation of a supramolecular structure at the base-acid molar ratio of 1:2 or higher.

Supramolecular structures are characterized by the additive and co-operative noncovalent interactions between the molecules.^37^ Hydrogen bond motifs have been used to form self-sorting supramolecular structures by their linear arrangement.^38^ The MIM-PFTB system does not involve multiple hydrogen bonds, but instead, it contains a special kind of SHB between the monoprotic weak acid and the monoprotic weak base, which exhibits the quantum mechanical property of hydrogen delocalization. These hydrogen bonds should be responsible for the supramolecular behavior of the MIM-PFTB mixtures, as they are the only noncovalent interactions present in the system. Thus, this study shows that quantum effects can drive the formation of solid structures when the right acid–base pairs are mixed, which has direct implications for supramolecular chemistry.

## Results and Discussion

The interactions between MIM and PFTB at different *r*_M_ give rise to a very interesting phase diagram (Figure 2a). Notably, mixtures of the two liquids at different molar ratios result in solids at room temperature with melting temperatures of 61.4, 27.0, and 20.3 °C for *r*_M_ of 1:2, 1:3, and 1:4, respectively. These melting points are significantly higher than the pure components of −18.8 °C for PFTB and −3.7 °C for MIM. In contrast, the melting points of other MIM-PFTB mixtures with *r*_M_ lower than 1:2 fall between those of the pure components, with the exception of the 2:1 complex, which has a melting point of −27.7 °C, likely due to the formation of a eutectic. While it is known that mixtures of acids and bases can form solids with high melting points, such as protic ionic liquids and supramolecular polymers,^39,40^ these systems typically involve acid–base reactions or the formation of hydrogen-bonded complexes with extended networks, enabled by multiple hydrogen bond donor groups in one or both molecules.^41^ In contrast, both the PFTB and MIM molecules have only one hydrogen bond donor and acceptor group, respectively. The complex phase diagram of MIM-PFTB mixtures reveals behavior that is clearly distinct from that of binary liquid mixtures of a monoprotic acid and a monoprotic base. Moreover, the liquid–solid phase transition of the supramolecular complexes is reversible. For example, when the solid complex with an *r*_M_ of 1:2 is heated above its melting point, it forms a liquid that solidifies when cooled down to room temperature. Overall, the results provide strong evidence for the supramolecular nature of the complex, which relies on noncovalent and reversible interactions to self-assemble into its solid form.

The nature of the noncovalent interaction is determined from the thermodynamics of the mixture as a function of the molar ratio of acid to base. The mixing of MIM and PFTB releases a large amount of heat, which is noticeable to the touch when preparing the samples. Calorimetric measurements reveal that the mixtures with *r*_M_ of 2:1, 1:1, and 1:2 exhibit distinct enthalpy changes of −52 ± 7, −56 ± 5, and −82 ± 6 kJ/mol, respectively. By comparison, the enthalpy of the reaction between a monoprotic base (NaOH) and a monoprotic acid (HCl) at 1:2, 2:1, and 1:1 molar ratios is found to be ∼52 kJ/mol (Table S1). While the latter case can be directly explained by the limiting reactant, the former case is more compelling because the addition of a second acid molecule further stabilizes the system, as evidenced by the larger enthalpy (−82 kJ/mol) when compared to the addition of a single acid molecule. While it is possible to attribute the enthalpy of the process resulting from the mixing of the acid and the base to a neutralization process, the difference in the enthalpy change observed when producing the 1:1 and the 1:2 *r*_M_ samples indicates the formation of two SHBs, as the addition of the second mole of PFTB leads to the cooperative effect commonly observed in hydrogen-bonded systems.^42,43^ Moreover, hydrogen-bonded complexes are known to be broken at high temperatures due to the large entropic penalty of the interaction.^44^ The formation of a complex stabilized by cooperative hydrogen bonds rather than by ionized species is in good agreement with the stoichiometry of the complex and its low melting point. In particular, MIM salts have 1:1 acid-to-base molar ratios and melting points well above room temperature, such as in the case of 1-methylimidazolium chloride and 1-methylimidazolium nitrate, with melting points of 75 and 70 °C for the 1:1 complexes, respectively.^45,46^

Hydrogen bonds typically have energies of less than 50 kJ/mol, with most interactions falling below 30 kJ/mol.^23^ Notably, one of the largest enthalpies of formation for an OH···N hydrogen bond is observed for picric acid and pyridazine, which is 32 kJ/mol.^47^ Conversely, SHBs have enthalpies of formation greater than 50 kJ/mol with some exceeding 100 kJ/mol.^23^ In the case of the 1:1 mixture of MIM and PFTB, the enthalpy of the hydrogen bond interaction is ∼50 kJ/mol for the formation of the first hydrogen bond and ∼30 kJ/mol for the second. The formation of a second hydrogen bond is not surprising, as it was previously observed in the MIM-HAc system that the second hydrogen bond further stabilizes the first hydrogen bond by facilitating the delocalization of the hydrogen atom between the acid and the base.^48^ PFTB in its liquid form is composed predominantly of monomers,^49^ whereas HAc is composed of dimers.^50^ While the strong self-association between acid molecules contributes to the stabilization of the delocalized hydrogen atom,^17^ it does not appear to promote the supramolecular behavior in the MIM-HAc system as their mixtures are all liquids at room temperature. Hence, it is likely that the weak self-association between PFTB molecules stabilizes the hydrogen atom delocalization in small complexes (dimers and trimers), which drives the formation of the MIM-PFTB supramolecular structure.

The calorimetric experiments demonstrate that MIM and PFTB form a molecular complex with SHBs. As shown in Figure 2b, the IR spectra are also consistent with the formation of a complex stabilized by hydrogen bonds. First, the IR spectra of the 1:1 and 1:2 *r*_M_ complexes do not present the signatures of the ionized state of MIM (i.e., MIMH^+^) as those appear in either the 1500–1600 cm^–1^ region for ring modes or the 3000–3200 cm^–1^ region for the N–H stretch (Figures 2 and S1). Second, IR spectroscopy shows the presence of a broad absorption band in the 2100–2800 cm^–1^ region for the 1:1 and 1:2 *r*_M_ mixtures, which is absent for the pure compounds (Figure 2b). The broad IR band observed for these complexes has been previously assigned to the signature of SHBs.^17^ Furthermore, the IR band of the MIM-PFTB complexes is red-shifted with respect to that of the MIM-HAc sample, suggesting the formation of a stronger hydrogen bond than in the previous case. Comparatively, pure methanol and a 1:1 mixture of MIM and methanol do not show the broad IR band centered at 2400 cm^–1^ (Figures 2 and S2), indicating that the broad band seen in the MIM-PFTB mixtures is not a signature of a conventional hydrogen-bonded complex.

The formation of an SHB in the complex is confirmed in the crystal structure of the 1-methylbenzimidazole (MBIM) and PFTB complex (Figure 2d), which, unlike the MIM-PFTB complex, is sufficiently stable to be measured via X-ray diffraction. The acid constant of MBIM is similar (p*K*_a_ = 5.55)^51^ to that of MIM, indicating that the presence of the benzene ring does not significantly affect the electron distribution and basicity of the imidazole group in the molecule, allowing us to extrapolate the results to MIM. The 1:1 MBIM-PFTB complex has a short hydrogen bond with a distance between nitrogen and oxygen atoms of 2.54 ± 0.01 Å (Figure 2d). As expected, the ATR-FTIR spectra of the 1:1 *r*_M_ for the complex form between MBIM and PFTB also show the broad IR band between 2100 and 2800 cm^–1^, which is not only consistent with the 1:1 MIM-PFTB complex (Figure 2b) but also directly relates the IR signature to the formation of an SHB.

In agreement with the IR spectroscopic results, the ^1^H NMR spectra show a large downfield shift for the hydroxyl proton in PFTB, which changes from 3 ppm in the pure PFTB to 14 ppm in the 1:1 mixture (Figure 2b). The large change in the chemical shift is in agreement with its large deshielding caused by delocalization of the acid hydrogen atom between the oxygen and nitrogen atoms.^17^ Note that the mixtures with *r*_M_ of 1:2 or above cannot be measured by ^1^H NMR due to their solid state. In contrast, the chemical shift of the MIM-MeOH samples shows a minimal change of less than 2 ppm for the hydrogen atom of the alcohol, which is consistent with the theoretical ^1^H NMR chemical shift of the hydroxyl hydrogen atom (see Supporting Information Table S4) and the downfield shift of common alcohols forming hydrogen bonds.^52,53^ However, the chemical shift produced by the alcohol is significantly smaller than that observed for the 1:1 MIM-PFTB complex (Figure 2b). Thus, the distinct IR and ^1^H NMR spectroscopic signatures of the MIM-PFTB complex strongly suggest the presence of an SHB with a delocalized hydrogen atom.

**Figure 3:**
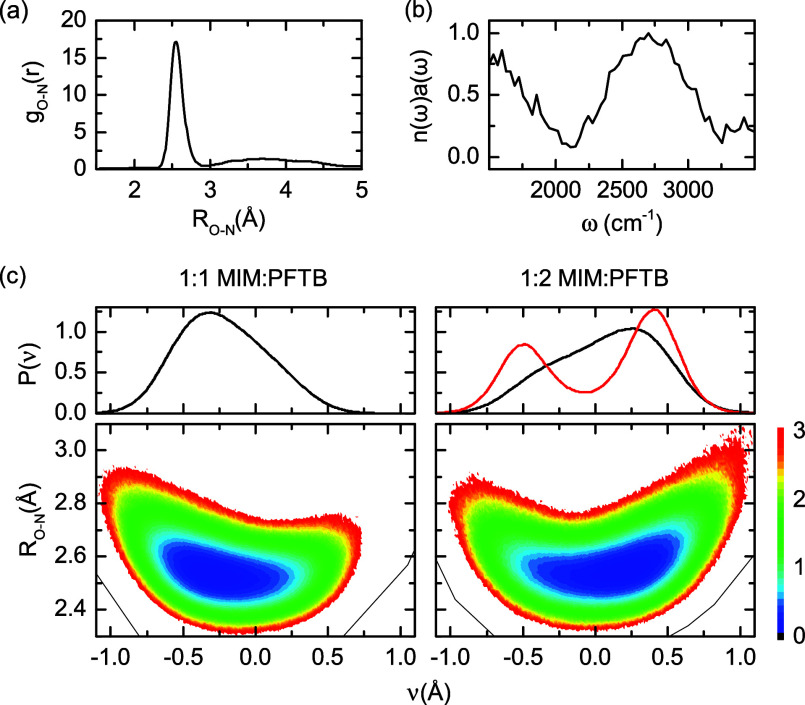
Structural and spectroscopic properties predicted from first-principles simulations. (a) O–N radial distribution functions for the 1:2 MIM-PFTB mixture from AI-PIMD simulations. (b) Simulated IR spectra for the 1:2 MIM-PFTB mixture from AI-RPMD simulations. (c) Probability distributions of the hydrogen atom position from AI-PIMD (black) and AIMD (red) and free energy surfaces for the shuttling of the hydrogen atom from AI-PIMD simulations. The proton sharing coordinate, ν = *d*_OH_–*d*_NH_, represents the position of the hydrogen atom in the hydrogen bond formed between MIM and PFTB.

First-principles simulations that incorporate the quantum mechanical nature of electrons and nuclei, including ab initio molecular dynamics (AIMD), ab initio path integral molecular dynamics (AI-PIMD), and ab initio thermostated ring polymer molecular dynamics simulations (AI-RPMD), confirm the formation of SHBs in the MIM-PFTB binary mixtures. From the AI-PIMD simulations of the 1:2 *r*_M_ mixture, the radial distribution functions between nitrogen and oxygen atoms indicate that MIM and PFTB form short and strong hydrogen bonds, according to their geometrical definition,^43^ with a donor–acceptor distance of ∼2.5 Å (Figure 3a), which is in excellent agreement with the O–N bond length measured in the X-ray structure of the MBIM-PFTB complex. The formation of an SHB in the complex is confirmed in the crystal structure of the 1-methylbenzimidazole (MBIM) and PFTB complex (Figure 2d) since it agrees with their geometrical definition.^43^ Furthermore, the computed IR spectra from AI-RPMD simulations confirm the broad absorption bands in the 2100–3000 cm^–1^ region, as observed experimentally (Figure 3b). Additionally, the theoretical ^1^H NMR chemical shift of the hydroxyl hydrogen atom in the 1:1 mixture of MIM and PFTB shows an 11 ppm change from the pure fluorinated alcohol (see Table S4), which is in full agreement with the experiments.

The formation of SHBs in the acid–base complex not only alters the position of the hydrogen atom within the participating pairs but also modifies the electronic structure of the components forming the complex. This effect is directly seen in the UV–vis spectra of the MIM-PFTB mixture (Figure 2c), which probes the lowest electronic state transitions of the molecular species. Interestingly, PFTB does not show any appreciable absorption within the 233 to 330 nm window. However, samples containing MIM, including MIM-PFTB, show a maximum peak near ∼240 nm and a very small one at ∼280 nm, which correspond to the allowed π → π* and nonallowed *n* → π* transitions, respectively.^54^ Both transitions are significantly modified by the presence of PFTB, as evidenced by the shift to lower wavelength of the π → π* transition and the disappearance of the *n* → π* one (Figure 2c). While the disappearance of the latter transition is related to the formation of the hydrogen bond, the effect seen in the former is not caused by the formation of a conventional hydrogen bond since the mixture of MIM and MeOH does not present any of the observed changes observed in the MIM-PFTB mixtures. Frontier molecular orbitals calculations reveal that the formation of the MIM-PFTB complex increases the energy gap between the highest occupied molecular orbital and the lowest unoccupied molecular orbital by 0.5 eV with respect to the 6.0 eV observed for pure MIM (see Table S5). This change in the HOMO–LUMO gap explains the shift to lower wavelengths observed for electronic excitations in the complex. Thus, the changes in the UV–vis spectra indicate the formation of an acid–base complex with an unconventional hydrogen bond, in agreement with the previous proposal that this type of bond should ultimately result in a change of the potential energy surface of the participating molecules as they become more covalent in nature when they are shortened.^24^

All the experimental data point to the formation of SHBs as the interactions behind the formation of the solid at room temperature for the 1:2 *r*_M_ mixture of MIM and PFTB. From the AI-PIMD simulations, it is found that the position of the hydrogen atom in the hydrogen bonds formed between MIM and PFTB depends significantly on the molar ratio between the acid and the base. In the 1:1 *r*_M_ mixture, the sample is predominantly composed of MIM-PFTB hydrogen-bonded dimers (see Figure S3). The corresponding two-dimensional free energy surface of the MIM-PFTB pair (Figure 3c) shows some degree of hydrogen atom delocalization with the proton shuttling coordinate, ν = *d*_OH_–*d*_NH_, having a considerable probability around ν = 0. However, the free energy of the dimer has a single minimum at ν = −0.27 Å, indicating that the hydrogen atom stays closer to the oxygen atom of PFTB. In contrast, the 1:2 *r*_M_ sample consists of both dimeric (MIM-PFTB) and trimeric (MIM-PFTB-PFTB) hydrogen-bonded complexes, with the latter being the dominant species (Figures S3 and S5). In these network structures, the hydrogen atom explores a wider phase space (Figure 3c), with the free energy minimum located at ν = 0.21 Å, suggesting that the hydrogen atom is located closer to the MIM (Figure 3c). Notably, the free energy associated with shuttling the hydrogen atom between the oxygen and nitrogen atoms in the trimer is less than 0.5 kcal/mol, demonstrating its quantum mechanical delocalization in the hydrogen bond. The changes in the preferred positions and movements of the hydrogen atom as a function of the acid concentration are clearly reflected in the corresponding probability distributions of ν (Figure 3c). It is important to note that hydrogen atom delocalization is only observed when nuclear quantum effects are explicitly considered, as the probability distributions of ν obtained from AIMD simulations show a double-well character corresponding to hydrogen atom transfer between MIM and PFTB (Figure 3c). This observation highlights the importance of nuclear quantum effects, in particular the zero-point energies of the O–H stretching modes, in determining the geometry and spectroscopic properties of these SHBs. Overall, first-principles simulations demonstrate the formation of SHBs between MIM and PFTB, in which the hydrogen atom is quantum mechanically delocalized. Furthermore, the simulations reveal how the geometry and length of the hydrogen bond networks influence the position of the hydrogen atom between the acid and the base, contributing enthalpically to the stabilization of these short interactions.

## Summary

This study shows the unconventional supramolecular chemistry of an acid–base pair due to both electronic and nuclear quantum effects of the hydrogen atom involved in the hydrogen bonds. This effect is described for mixtures of MIM and PFTB, which form solid supramolecular complexes at room temperature when the base-acid molar ratio is greater than 1:2. The IR and ^1^H NMR spectra display the signature of strong, but thermally labile, hydrogen bonds in the solid complex. The SHBs are confirmed via the X-ray structure between PFTB and a MIM analog. In addition, the ^1^H NMR chemical shift of the acidic proton provides direct evidence for its delocalization. First-principles simulations correctly predict the formation of SHBs between MIM and PFTB and demonstrate that their uniqueness is partly due to the delocalization of the hydrogen atom. These unconventional hydrogen bonds are fully consistent with the supramolecular character of the complex and its poor thermal stability. These findings highlight that quantum delocalization of the hydrogen atom is fundamental to the stability and unusual thermal properties of MIM-PFTB hydrogen-bonded complexes. Overall, the formation of the MIM-PFTB supramolecular structure clearly suggests a potential new mechanism in supramolecular chemistry, in which quantum mechanical effects play a decisive role in hydrogen bond interactions.

## Supporting Information Available

Details on experimental and simulation methods, MDSC thermograms, ATR-FTIR spectra, ^1^HNMR data, crystallographic data, HOMO–LUMO energies, and probability distribution of hydrogen bond networks (PDF)
