# Supplementary material for: Quantum Mechanical Behavior of Hydrogen Bonds Enables Supramolecular Structure in a Weak Acid–Base Monoprotic Complex
Source: J Am Chem Soc. 2025 Apr 9;147(16):13251–7. doi: 10.1021/jacs.4c17870 (PMC12022972; doi:10.1021/jacs.4c17870)
Supplement: Supplementary file 1 — ja4c17870_si_001.pdf [file ja4c17870_si_001.pdf]

Supplemental Information

Quantum Mechanical Behavior of Hydrogen Bonds Enables Supramolecular Structure in a Weak Acid-Base Monoprotic Complex

Anit Gurung[1†], Rui Zhang[2†], Lu Wang[2*] and Daniel Kuroda[1*]

[1] *Department of Chemistry, Louisiana State University, Baton Rouge, LA, 70803*

[2] *Department of Chemistry and Chemical Biology, Institute for Quantitative Biomedicine, Rutgers University, Piscataway, NJ, 08854*

Table of Contents

1. Experimental Methods 2
   1.1 Sample Preparation 2
   1.2 Linear Infrared Spectroscopy 2
   1.3 ¹H-NMR spectroscopy 2
   1.4 Ultraviolet-Visible Spectroscopy 2
   1.5 Single Crystal X-ray Diffraction 2
   1.6 Modulated DSC 3
   1.7 Calorimetric Measurements 3
   1.8 First-Principles Simulations 3
   1.9 DFT calculations 4
   1.10 Molecular dynamics simulations 5
2. Tables 6
3. Figures 8
References

1. Experimental Methods

1.1 Sample Preparation

1-Methylimidazole (99% pure, Beantown Chemical), perfluoro-tert-butanol (98%, Oakwood Chemical), 1-methylbenzimidazole (99% pure, AmBeed), and methanol (99.8% pure, VWR Chemicals) were used. The liquid chemicals were dried over 4 A molecular sieves for 24 hours. The binary mixtures were prepared by mixing the acid and the base at different molal compositions. The drying and sample preparation were all performed in a nitrogen-filled glove box to minimize water contamination. The 1-methylimidazolium nitrate salt was prepared by the neutralization reaction of 1-methylimidazole with nitric acid in aqueous solution, from which water is removed under vacuum at 40°C for 12 hours.[1]

1.2 Linear Infrared Spectroscopy

The linear infrared (IR) spectra of the samples were obtained using a Bruker Tensor 27 FTIR spectrometer equipped with a liquid nitrogen-cooled, narrow-band mercury cadmium telluride detector. The resolution of the FTIR was 0.5 cm^{-1}, and each spectrum was obtained by averaging 40 scans. Samples were placed between two 2 mm CaF_2 windows, separated by Teflon spacers of different thicknesses. The FTIR sample cells were prepared in a nitrogen-filled glove box. Attenuated total reflection (ATR)-FTIR spectra were measured on a Bruker Alpha spectrometer equipped with a room-temperature DLATGS detector, a mid-IR source (4000 to 400 cm^{-1}), a KBr beamsplitter and a platinum attenuated total reflectance (ATR) accessory with a diamond crystal plate. All ATR data were collected as the average of over 16 scans with a minimum resolution of 1 cm^{-1}.

1.3 ^{1}H-NMR spectroscopy

The ^{1}H-NMR spectra were collected on a 9.4 T (400.13 MHz ^{1}H resonance frequency) Bruker AVANCE III HD NanoBay spectrometer with Ascend magnet. All the spectra of liquid samples were collected with 16 scans and deuterated DMSO in a capillary tube is used for locking signal, except for the $MIMH^{+}[NO_3]^{-}$ that was dissolved in $CDCl_3$

1.4 Ultraviolet-Visible Spectroscopy

The UV-Vis spectra were collected using Agilent 8453 UV/Vis/NIR spectrophotometer (190 to 1100 nm) equipped with a 7-cell accessory and a VWR 1167 circulating bath for temperature control. Samples were dissolved in dichloromethane (DCM) to prepare 0.1 M solutions and placed in 0.5 ml quartz cuvettes with a path length of 1cm. UV-Vis measurements were carried out at wavelengths from 190 to 750 nm with 1nm intervals.

1.5 Single Crystal X-ray Diffraction

Single crystal X-ray diffraction was performed using a Bruker D8 Venture DUO diffractometer equipped with two X-ray sources (Cu or Ag) and a Photon III C14 detector equipped with an Oxford Cryostream model 1000 cryostat. A Zeiss 2000-C stereo zoom polarizing microscope for sample manipulation and crystal mounting. The crystal was grown by dissolving MBIM/PFTB solid in dichloromethane (DCM) followed by slow evaporation of DCM. A summary of the single crystal X-ray data collection and structural refinement can be found in Table S2.

1.6 Modulated DSC

The melting point of the samples (see Figure S4) were determined using a TA Instruments Discovery DSC250 Modulated Differential Scanning Calorimeter (MDSC) with refrigerated system cooling (RSC-90) from -90°C to 550°C. 5-20 mg of samples was placed into hermetically sealed Alodined pans, which were prepared inside the nitrogen-filled glove box.

1.7 Calorimetric Measurements

The enthalpy of formation was determined using a double-wall calorimeter consisting of an inner aluminum reservoir of 150 ml capacity, an outer aluminum case, a plastic insulating ring and a polystyrene lining. The calorimetric constant was determined by adding 40 ml of deionized water at high temperature. The amount of deionized water was kept constant for all the measurements. A digital thermometer probe with a 0.1 ºC resolution was immersed in the deionized water and the change in water temperature was recorded and used to evaluate the heat transfer.

1.8 First-Principles Simulations

AIMD and AI-PIMD simulations were performed for MIM-PFTB mixtures at r_M of 1:1 and 1:2 using the CP2K and i-PI programs.[2, 3] The initial structures were constructed by randomly mixing MIM and PFTB molecules in the appropriate molecular ratios using the Packmol package.[4] The resulting 1:1 and 1:2 systems comprised 62 and 60 molecules, respectively, organized in a cubic box with a side length of approximately 22 Å, as summarized in Table S3. The simulations were performed under the NVT condition at 297 K. The electronic structures were described by the revPBE density functional with the D3 dispersion corrections,[5-7] and the core electrons were represented by the Goedecker-Teter-Hutter pseudopotentials.[8] Kohn-Sham orbitals were expanded in a DZVP atom-centered basis set with a cutoff of 300 Ry for the valence charge density. Note that, due to the limited time scales of the first-principles simulations, the 1:1 and 1:2 mixtures all remained in the liquid phase. Although the solid phase of the 1:2 MIM-PFTB mixture could not be reproduced, the simulations are still expected to offer valuable insights into the key interactions in the system.

For the AIMD simulations, each system underwent a 10-ps equilibration, followed by a 200-ps production run using a multiple time-step method.[9-11] Using the reversible reference system propagator approach to the MTS integration, we applied a time step of 2 fs for the full force evaluations from DFT calculations and a time step of 0.5 fs for the reference forces, computed via the self-consistent charge density-functional tight-binding (SCC-DFTB3) method[12] with the 3ob parameter set applied for all atoms.[13, 14] A global stochastic velocity rescaling thermostat was applied with a time constant of 1 ps to control the temperature.[15] For AI-PIMD simulations, each atom was represented by 6 ring polymer beads using the path integral generalized Langevin equation approach.[16] Following a 5-ps equilibration, a 50-ps production run was performed for each system.

In addition, AI-TRPMD simulations were conducted for the MIM-PFTB mixture at 1:2 molar ratio. TRPMD was applied to remove spurious resonances in the IR spectra produced by regular RPMD simulations.[17, 18] TRPMD simulations utilize the isomorphism between a quantum mechanical system and a classical system of ring polymers, where each atom is represented by *32* beads

connected by harmonic springs.[17, 19-21] A global path integral Langevin equation (PILE-G) thermostat was applied to control the simulation temperature at 297 K.[22] Initial configurations were taken from the AI-PIMD simulations, and the AI-TRPMD trajectory spanned 20 ps for the calculation of the IR spectra. A time step of 0.5 fs was used for both the AI-PIMD and the AI-TRPMD simulations.

Based on the AI-TRPMD simulations, the IR spectra of a system can be calculated from the Fourier transform of the quantum mechanical dipole-dipole time correlation function,[17, 18, 23-27]

$$n(\omega)\alpha(\omega) = \frac{\pi\omega}{3\hbar c V \epsilon_0}\left(1 - e^{-\beta\hbar\omega}\right) I(\omega) \quad (1)$$

where

$$I(\mathrm{w}) = \frac{1}{2\pi}\int_{-\infty}^{\infty} dt e^{-i\omega t}\langle \vec{\mu}(0)\cdot\vec{\mu}(t)\rangle \quad (2)$$

Equation 1 can also be written as

$$n(\omega)\alpha(\omega) = \frac{\pi\beta\omega^2}{3cV\epsilon_0}\tilde{I}(\omega) \quad (3)$$

where $\tilde{I}(\omega)$ is the Fourier transform of the Kubo-transformed dipole-dipole time correlation function,[17, 18, 23-26]

$$\tilde{I}(\mathrm{w}) = \frac{1-e^{-\beta\hbar\omega}}{\beta\hbar\omega} I(\omega) = \frac{1}{2\pi}\int_{-\infty}^{\infty} dt e^{-i\omega t}\tilde{C}(t) \quad (4)$$

and

$$\tilde{C}(t) = \frac{1}{\beta}\int_0^\beta d\lambda \langle \vec{\boldsymbol{\mu}}(-i\lambda\hbar)\cdot\vec{\boldsymbol{\mu}}(t)\rangle \quad (5)$$

In equation 5, β is the inverse temperature, i is the imaginary unit, λ is an imaginary time variable, ħ is the reduced Planck constant, and $\vec{\boldsymbol{\mu}}(t)$ is the molecular dipole moment. The angular brackets represent statistical averages. TRPMD simulations provide the approximate Kubo-transformed time-correlation function,[28-30]

$$\tilde{C}(t) = \langle \vec{\boldsymbol{\mu}}_c(0)\cdot\vec{\boldsymbol{\mu}}_c(t)\rangle \quad (6)$$

where $\vec{\boldsymbol{\mu}}_c(t)$ is the dipole moment of a molecule at time *t* averaged over the P ring polymer beads,

$$\vec{\boldsymbol{\mu}}_c(t) = \frac{1}{P}\sum_{i=1}^{P}\vec{\boldsymbol{\mu}}_i(t) \quad (7)$$

The molecular dipole moment for the ith bead, $\vec{\boldsymbol{\mu}}_i(t)$, was obtained from the maximally localized Wannier function scheme using the CP2K program.[31, 32] From the AI-TRPMD trajectories, 10,000 frames were extracted at a time step of 2 fs to compute the IR spectra from equation 3.

1.9 DFT calculations

DFT calculations were performed to obtain the ^{1}H NMR chemical shifts and frontier orbitals for various systems using the Gaussian 16 package.[33] The electronic structure was described using the B3LYP density functional, the D3 dispersion correction, and the 6-311++G(d,p) basis set.[7, 34, 35]

For NMR calculations, we optimized the geometries of the PFTB monomer and the MIM-PFTB and MIM-MeOH hydrogen-bonded dimers and obtained the isotropic magnetic shielding tensors using the gauge-independent atomic orbital (GIAO) approach.[36] Chemical shifts were calculated using tetramethylsilane (TMS) as the reference. All calculations were performed in carbon tetrachloride, which was described by the polarizable continuum model (PCM) with a dielectric constant of 2.24.[37] For the frontier orbital calculations, the HOMO and LUMO orbitals and energies were computed from the optimized the geometries of PFTB, MIM and MeOH monomers, as well as MIM-PFTB and MIM-MeOH hydrogen-bonded dimers.

1.10 Molecular dynamics simulations

Classical molecular dynamics (MD) simulations were performed to determine the density of MIM-PFTB mixtures using the AMBER 2020 software.[38] Using the Packmol package[4], we randomly mixed a total of 900 MIM and PFTB molecules within a cubic box with a side length of 60 Å, generating mixtures with specified MIM:PFTB ratios of 1:1 and1:2. Both MIM and PFTB were modeled using the second-generation General Amber Force Field (GAFF2).[39] Each system underwent energy minimization, first using the steepest descent algorithm for 500 cycles, followed by the conjugate gradient algorithm for 5,000 cycles. The systems were then gradually heated from 0 to 298 K over a duration of 100 ps. Equilibration was carried out at a constant temperature of 298 K and pressure of 1 atm for 1 ns. Production runs were conducted for 10 ns in the NPT ensemble, with trajectories saved every 1 ps for further analysis. A 2-fs time step was used for all simulations. Temperature control was achieved using the Langevin thermostat with a collision frequency of 2.0 ps^{-1}, and pressure was regulated with the Berendsen barostat, with a relaxation time of 1 ps.[40, 41] Periodic boundary conditions were applied, and chemical bonds involving hydrogen atoms were constrained using the SHAKE algorithm.[42] We treated long-range electrostatic interactions using the particle-mesh Ewald method,[43] and applied a 10 Å cutoff for van der Waals interactions. All systems remained in the liquid phase during the MD simulations.

Density calculations were based on the last 2 ns of the production runs. The determined density was 1.47 g/mL for the 1:1 mixture, closely matching experimental results of 1.48 g/ml. The density for the 1:2 mixture was 1.54 g/mL from MD simulations. Experimental data for this system is unavailable as the sample is in the solid state under room temperature. The densities obtained from the MD simulations were used to calculate the box sizes for the first-principles simulations.

2. Tables

Table S1. Reaction enthalpy between 1M NaOH(aq) and 1M HCl(aq) at r_M: 2:1, 1:1 and 1:2.

**NaOH:HCl**	**-ΔH (kJ/ mol)**
2:1	53 ± 1
1:1	52 ± 1
1:2	52 ± 1

Table S2. Single crystal X-ray data collection and structural refinement for 1:1 MBIM-PFTB crystal

Crystal Name	**1:1 MBIM_PFTB**
Empirical Formula	$C_8H_8N_2 \cdot C_4HF_9O$
Temperature (K)	100
Formula Weight (g/mol)	368.21
Radiation	Cu *K*α radiation, λ = 1.54184 Å
Space group	Triclinic, $P\bar{1}$
*a (*Å)	6.8326 (11)
*b (*Å)	10.0921 (16)
*c (*Å)	21.204 (4)
α (°)	89.853 (12)
β (°)	83.272 (12)
γ (°)	75.947 (11)
Volume (Å^3)	1408.1 (4)
Size (mm^3)	0.11 × 0.07 × 0.02
ρ_{calc} (g/cm^3)	1.737
μ (mm^{-1})	1.77
Z	4
F (000)	736
θ (°)	4.9 to 61.8
Measured Reflections	36704
Independent Reflections	4887
Refinement on F^2	H-atom parameters not defined
Goodness-of-fit on F^2	1.026
Final R indexes [$F^2 > 2\sigma(F^2)$]	R = 0.0910<br>wR = 0.2712
Final R indexes [*Full Data*]	R = 0.1332<br>wR = 0.2403

Table S3. Summary of system size for the first-principles simulations

r_M	Box length (Å)	Number of MIM	Number of PFTB
1:1	22.3	31	31
1:2	22.9	20	40

Table S4. Calculated and experimental ^{1}H NMR chemical shifts for the acidic proton in PFTB across various systems. The calculated chemical shifts are obtained from DFT evaluations of monomeric or dimeric structures in DMSO solutions.

	δ_H^{DFT} (ppm)	$\delta_H^{\boldsymbol{exp}}$ (ppm)
PFTB	2.7	3.1
1:1 MIM-PFTB dimer	13.9	14.1
1:1 MIM-MeOH dimer	6.6	5.6

Table S5. HOMO and LUMO energies and energy gaps from DFT calculations of gas-phase monomer or dimer systems.

	E_{HOMO} (ev)	E_{LUMO} (eV)	Energy Gap (eV)
PFTB	-9.94	-0.74	9.20
MIM	-6.42	-0.44	5.98
MeOH	-7.74	-0.36	7.38
1:1 MIM-PFTB dimer	-7.24	-0.73	6.51
1:1 MIM-MeOH dimer	-6.53	-0.60	5.93

3. Figures

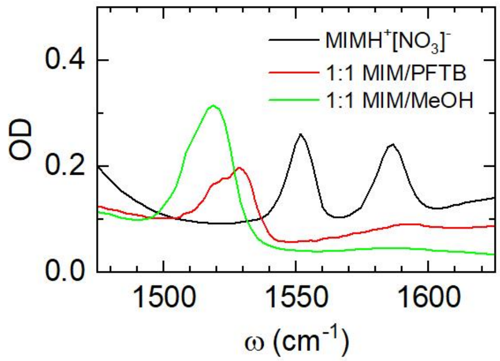

Figure S1. ATR-FTIR spectral region between 1500 and 1600 cm^{-1} of $MIMH^{+}[NO_3]^{-}$ (black line), 1:1 MIM/PFTB (red line) and MIM/MeOH (green line).

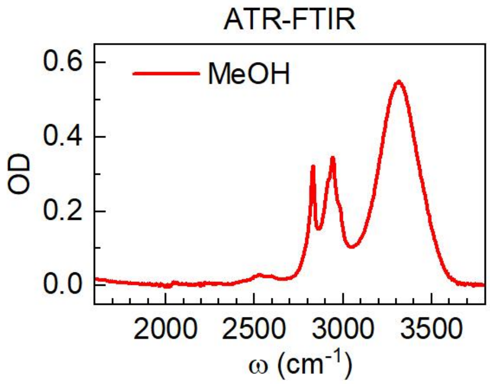

Figure S2. ATR-FTIR spectrum of pure methanol (MeOH).

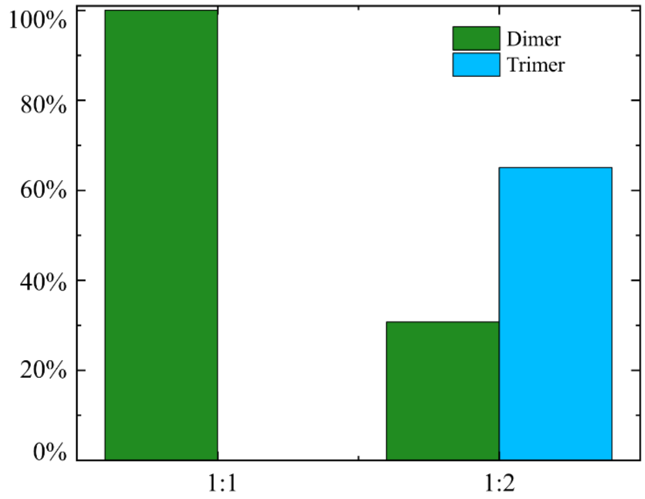

Figure S3. Probability distributions of hydrogen bond networks from AI-PIMD simulations of the 1:1 and 1:2 MIM-PFTB mixtures. In the 1:1 mixture, hydrogen bonding is observed exclusively between MIM and PFTB molecules. In the 1:2 mixture, approximately 4% of hydrogen bonds occur between PFTB molecules.

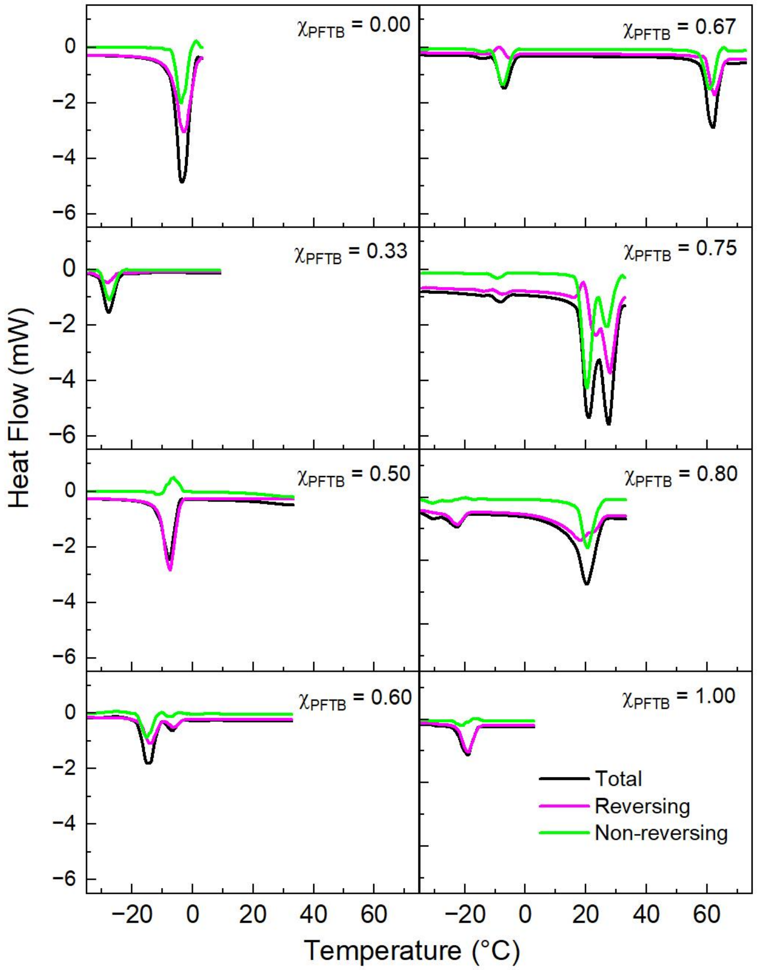

Figure S4. MDSC thermograms of MIM/PFTB complexes at varying molar compositions, where χ_{PFTB} = 0, 0.33, 0.5, 0.6, 0.67, 0.75, 0.8 and 1

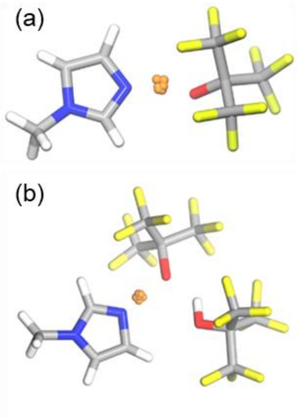

Figure S5. Representative structures of hydrogen bonded (a) dimer and (b) trimer complexes of PFTB and MIM. Silver, red, blue, yellow, and white represent C, O, N, F and H atoms, respectively, and the orange spheres represent the ring polymer beads of the delocalized hydrogen atom.

References

(1) Smiglak, M.; Hines, C. C.; Reichert, W. M.; Vincek, A. S.; Katritzky, A. R.; Thrasher, J. S.; Sun, L. Y.; McCrary, P. D.; Beasley, P. A.; Kelley, S. P.; et al. Synthesis, limitations, and thermal properties of energetically-substituted, protonated imidazolium picrate and nitrate salts and further comparison with their methylated analogs. *New J Chem* **2012**, *36* (3), 702-722. DOI: 10.1039/c1nj20677j.

(2) Kapil, V.; Rossi, M.; Marsalek, O.; Petraglia, R.; Litman, Y.; Spura, T.; Cheng, B.; Cuzzocrea, A.; Meißner, R. H.; Wilkins, D. M.; et al. i-PI 2.0: A universal force engine for advanced molecular simulations. *Computer Physics Communications* **2019**, *236*, 214-223. DOI: https://doi.org/10.1016/j.cpc.2018.09.020.

(3) Kühne, T. D.; Iannuzzi, M.; Del Ben, M.; Rybkin, V. V.; Seewald, P.; Stein, F.; Laino, T.; Khaliullin, R. Z.; Schütt, O.; Schiffmann, F.; et al. CP2K: An electronic structure and molecular dynamics software package - Quickstep: Efficient and accurate electronic structure calculations. *The Journal of Chemical Physics* **2020**, *152* (19), 194103. DOI: 10.1063/5.0007045.

(4) Martínez, L.; Andrade, R.; Birgin, E. G.; Martínez, J. M. PACKMOL: A package for building initial configurations for molecular dynamics simulations. *Journal of Computational Chemistry* **2009**, *30* (13), 2157-2164. DOI: https://doi.org/10.1002/jcc.21224.

(5) Perdew, J. P.; Burke, K.; Ernzerhof, M. Generalized Gradient Approximation Made Simple. *Physical Review Letters* **1996**, *77* (18), 3865-3868.

(6) Zhang, Y.; Yang, W. Comment on ``Generalized Gradient Approximation Made Simple''. *Physical Review Letters* **1998**, *80* (4), 890-890. DOI: 10.1103/PhysRevLett.80.890.

(7) Grimme, S.; Antony, J.; Ehrlich, S.; Krieg, H. A consistent and accurate ab initio parametrization of density functional dispersion correction (DFT-D) for the 94 elements H-Pu. *The Journal of Chemical Physics* **2010**, *132* (15), 154104. DOI: doi:http://dx.doi.org/10.1063/1.3382344.

(8) Goedecker, S.; Teter, M.; Hutter, J. Separable dual-space Gaussian pseudopotentials. *Physical Review B* **1996**, *54* (3), 1703-1710. DOI: 10.1103/PhysRevB.54.1703.

(9) Tuckerman, M.; Berne, B. J.; Martyna, G. J. Reversible multiple time scale molecular dynamics. *The Journal of Chemical Physics* **1992**, *97* (3), 1990-2001. DOI: 10.1063/1.463137.

(10) Luehr, N.; Markland, T. E.; Martínez, T. J. Multiple time step integrators in ab initio molecular dynamics. *The Journal of Chemical Physics* **2014**, *140* (8). DOI: 10.1063/1.4866176.

(11) Kapil, V.; VandeVondele, J.; Ceriotti, M. Accurate molecular dynamics and nuclear quantum effects at low cost by multiple steps in real and imaginary time: Using density functional theory to accelerate wavefunction methods. *The Journal of Chemical Physics* **2016**, *144* (5). DOI: 10.1063/1.4941091.

(12) Gaus, M.; Cui, Q.; Elstner, M. DFTB3: Extension of the Self-Consistent-Charge Density-Functional Tight-Binding Method (SCC-DFTB). *Journal of Chemical Theory and Computation* **2011**, *7* (4), 931-948. DOI: 10.1021/ct100684s.

(13) Gaus, M.; Goez, A.; Elstner, M. Parametrization and Benchmark of DFTB3 for Organic Molecules. *Journal of Chemical Theory and Computation* **2013**, *9* (1), 338-354. DOI: 10.1021/ct300849w.

(14) Kubillus, M.; Kubař, T.; Gaus, M.; Řezáč, J.; Elstner, M. Parameterization of the DFTB3 Method for Br, Ca, Cl, F, I, K, and Na in Organic and Biological Systems. *Journal of Chemical Theory and Computation* **2015**, *11* (1), 332-342. DOI: 10.1021/ct5009137.

(15) Bussi, G.; Donadio, D.; Parrinello, M. Canonical sampling through velocity rescaling. *J Chem Phys* **2007**, *126* (1), 014101. DOI: 10.1063/1.2408420.

(16) Ceriotti, M.; Bussi, G.; Parrinello, M. Langevin Equation with Colored Noise for Constant-Temperature Molecular Dynamics Simulations. *Physical Review Letters* **2009**, *102* (2), 020601. DOI: 10.1103/PhysRevLett.102.020601.

(17) Rossi, M.; Ceriotti, M.; Manolopoulos, D. E. How to remove the spurious resonances from ring polymer molecular dynamics. *The Journal of Chemical Physics* **2014**, *140* (23). DOI: 10.1063/1.4883861.
(18) Rossi, M.; Liu, H.; Paesani, F.; Bowman, J.; Ceriotti, M. Communication: On the consistency of approximate quantum dynamics simulation methods for vibrational spectra in the condensed phase. *The Journal of Chemical Physics* **2014**, *141* (18). DOI: 10.1063/1.4901214.
(19) Marsalek, O.; Markland, T. E. Quantum Dynamics and Spectroscopy of Ab Initio Liquid Water: The Interplay of Nuclear and Electronic Quantum Effects. *The Journal of Physical Chemistry Letters* **2017**, *8* (7), 1545-1551. DOI: 10.1021/acs.jpclett.7b00391.
(20) Ruiz Pestana, L.; Marsalek, O.; Markland, T. E.; Head-Gordon, T. The Quest for Accurate Liquid Water Properties from First Principles. *The Journal of Physical Chemistry Letters* **2018**, *9* (17), 5009-5016. DOI: 10.1021/acs.jpclett.8b02400.
(21) Chandler, D.; Wolynes, P. G. Exploiting the Isomorphism between Quantum-Theory and Classical Statistical-Mechanics of Polyatomic Fluids. *Journal of Chemical Physics* **1981**, *74* (7), 4078-4095. DOI: 10.1063/1.441588.
(22) Ceriotti, M.; Parrinello, M.; Markland, T. E.; Manolopoulos, D. E. Efficient stochastic thermostatting of path integral molecular dynamics. *The Journal of Chemical Physics* **2010**, *133* (12). DOI: 10.1063/1.3489925.
(23) Habershon, S.; Manolopoulos, D. E.; Markland, T. E.; Miller, T. F. Ring-Polymer Molecular Dynamics: Quantum Effects in Chemical Dynamics from Classical Trajectories in an Extended Phase Space. *Annu Rev Phys Chem* **2013**, *64*, 387-413. DOI: 10.1146/annurev-physchem-040412-110122.
(24) Habershon, S.; Fanourgakis, G. S.; Manolopoulos, D. E. Comparison of path integral molecular dynamics methods for the infrared absorption spectrum of liquid water. *Journal of Chemical Physics* **2008**, *129* (7). DOI: 10.1063/1.2968555.
(25) Craig, I. R.; Manolopoulos, D. E. Quantum statistics and classical mechanics: Real time correlation functions from ring polymer molecular dynamics. *Journal of Chemical Physics* **2004**, *121* (8), 3368-3373. DOI: 10.1063/1.1777575.
(26) Kubo, R. Statistical-Mechanical Theory of Irreversible Processes .1. General Theory and Simple Applications to Magnetic and Conduction Problems. *J Phys Soc Jpn* **1957**, *12* (6), 570-586. DOI: 10.1143/Jpsj.12.570.
(27) Zwanzig, R. *Nonequilibrium statistical mechanics*; Oxford University Press, 2001.
(28) Witt, A.; Ivanov, S. D.; Shiga, M.; Forbert, H.; Marx, D. On the applicability of centroid and ring polymer path integral molecular dynamics for vibrational spectroscopy. *Journal of Chemical Physics* **2009**, *130* (19). DOI: 10.1063/1.3125009.
(29) Yu, Q.; Bowman, J. M. Classical, Thermostated Ring Polymer, and Quantum VSCF/VCI Calculations of IR Spectra of $H_7O_3^+$ and $H_9O_4^+$ (Eigen) and Comparison with Experiment. *J Phys Chem A* **2019**, *123* (7), 1399-1409. DOI: 10.1021/acs.jpca.8b11603.
(30) Inakollu, V. S. S.; Yu, H. B. Comparative studies of IR spectra of deprotonated serine with classical and thermostated ring polymer molecular dynamics simulations. *Struct Dynam-Us* **2021**, *8* (5). DOI: 10.1063/4.0000124.
(31) Marzari, N.; Vanderbilt, D. Maximally localized generalized Wannier functions for composite energy bands. *Physical Review B* **1997**, *56* (20), 12847-12865. DOI: 10.1103/PhysRevB.56.12847.
(32) Thomas, M.; Brehm, M.; Fligg, R.; Vöhringer, P.; Kirchner, B. Computing vibrational spectra from ab initio molecular dynamics. *Phys Chem Chem Phys* **2013**, *15* (18), 6608-6622, 10.1039/C3CP44302G. DOI: 10.1039/C3CP44302G.
(33) *Gaussian 16 Rev. A.03*; Wallingford, CT, 2016.
(34) Becke, A. D. Density‐functional thermochemistry. III. The role of exact exchange. *The Journal of Chemical Physics* **1993**, *98* (7), 5648-5652. DOI: 10.1063/1.464913.

(35) Lee, C.; Yang, W.; Parr, R. G. Development of the Colle-Salvetti correlation-energy formula into a functional of the electron density. *Phys Rev B Condens Matter* **1988**, *37* (2), 785-789. DOI: 10.1103/physrevb.37.785.
(36) Ditchfield, R. Self-consistent perturbation theory of diamagnetism. *Molecular Physics* **1974**, *27* (4), 789-807. DOI: 10.1080/00268977400100711.
(37) Cancès, E.; Mennucci, B.; Tomasi, J. A new integral equation formalism for the polarizable continuum model: Theoretical background and applications to isotropic and anisotropic dielectrics. *The Journal of Chemical Physics* **1997**, *107* (8), 3032-3041. DOI: 10.1063/1.474659.
(38) Case, D. A.; Belfon, K.; Ben-Shalom, I. Y.; Brozell, S. R.; Cerutti, D. S.; III, T. E. C.; Cruzeiro, V. W. D.; Darden, T. A.; Duke, R. E.; Giambasu, S.; et al. *AMBER 2020*; University of California, San Francisco, 2020.
(39) Wang, J.; Wolf, R. M.; Caldwell, J. W.; Kollman, P. A.; Case, D. A. Development and testing of a general amber force field. *J Comput Chem* **2004**, *25* (9), 1157-1174. DOI: 10.1002/jcc.20035.
(40) Rosenberg, R. O.; Boughaleb, Y.; Nitzan, A.; Ratner, M. A. Effective potentials from Langevin dynamic simulations of framework solid electrolytes. *Solid State Ionics* **1986**, *18-19*, 127-135. DOI: https://doi.org/10.1016/0167-2738(86)90099-8.
(41) Berendsen, H. J. C.; Postma, J. P. M.; van Gunsteren, W. F.; DiNola, A.; Haak, J. R. Molecular dynamics with coupling to an external bath. *The Journal of Chemical Physics* **1984**, *81* (8), 3684-3690. DOI: 10.1063/1.448118.
(42) Miyamoto, S.; Kollman, P. A. Settle: An analytical version of the SHAKE and RATTLE algorithm for rigid water models. *Journal of Computational Chemistry* **1992**, *13* (8), 952-962. DOI: https://doi.org/10.1002/jcc.540130805.
(43) Darden, T.; York, D.; Pedersen, L. Particle mesh Ewald: An N·log(N) method for Ewald sums in large systems. *The Journal of Chemical Physics* **1993**, *98* (12), 10089-10092. DOI: 10.1063/1.464397.
